# Responses of Vascular Endothelial Cells to Photoembossed Topographies on Poly(Methyl Methacrylate) Films

**DOI:** 10.3390/jfb7040033

**Published:** 2016-12-09

**Authors:** Lin Qiu, Nanayaa F. Hughes-Brittain, Cees W. M. Bastiaansen, Ton Peijs, Wen Wang

**Affiliations:** School of Engineering and Materials Science, Queen Mary University of London, Mile End Road, London E1 4NS, UK; helen-qiu02@hotmail.com (L.Q.); nanayaah @hotmail.com (N.F.H.-B.); c.w.m.bastiaansen@qmul.ac.uk (C.W.M.B.)

**Keywords:** surface texture, polymer, photoembossing, cell interaction, cell proliferation and migration

## Abstract

Failures of vascular grafts are normally caused by the lack of a durable and adherent endothelium covering the graft which leads to thrombus and neointima formation. A promising approach to overcome these issues is to create a functional, quiescent monolayer of endothelial cells on the surface of implants. The present study reports for the first time on the use of photoembossing as a technique to create polymer films with different topographical features for improved cell interaction in biomedical applications. For this, a photopolymer is created by mixing poly(methyl methacrylate) (PMMA) and trimethylolpropane ethoxylate triacrylate (TPETA) at a 1:1 ratio. This photopolymer demonstrated an improvement in biocompatibility over PMMA which is already known to be biocompatible and has been extensively used in the biomedical field. Additionally, photoembossed films showed significantly improved cell attachment and proliferation compared to their non-embossed counterparts. Surface texturing consisted of grooves of different pitches (6, 10, and 20 µm) and heights (1 µm and 2.5 µm). The 20 µm pitch photoembossed films significantly accelerated cell migration in a wound-healing assay, while films with a 6 µm pitch inhibited cells from detaching. Additionally, the relief structure obtained by photoembossing also changed the surface wettability of the substrates. Photoembossed PMMA-TPETA systems benefited from this change as it improved their water contact angle to around 70°, making it well suited for cell adhesion.

## 1. Introduction

The major problem in vascular grafts, especially those with diameters of less than 4 mm, is thrombus and neointima formation [1,2,3]. A promising approach to overcome these issues is to create a functional, quiescent monolayer of endothelial cells on the surface of implants [1,2]. In this way, cell adhesion to the extracellular matrix (ECM), morphogenesis, immunity, and wound healing may be influenced [3,4]. The vascular endothelium also controls the transport of substances from the blood into the vessel wall [5,6,7]. The destruction of this control can cause cardiovascular disease [8]. Therefore, to understand and accelerate the healing of the endothelium after wounding it is important to avoid the beginning of focal disease [7]. For a polymer scaffold, endothelial cell adhesion and proliferation are both affected by chemical constitution and roughness of the surface. Previous studies have shown that grooved textures with depths exceeding 500 nm result in stronger cell interactions [9]. Surface topography has also been shown to incite changes in the morphology and orientation of adherent endothelial cells [2,10,11]. In the meantime, cell shape, adhesion, and migration [7,12] can be affected by flow beyond certain shear stresses under dynamic circulation similar to circulating blood.

Smooth surfaces with no relief structures of biomaterials are known to promote inflammation whereas textured surfaces with ridges or pillars, for example, have proven to be less of a problem [13]. Topography can be defined as the morphology of a surface. Harrison [14] first reported the migration of cultured cells that were guided by spider webs. This discovery was later supported by other researchers [15,16]. The term ‘contact guidance’ was introduced by Weiss [17] to describe cell orientation and cell migration in response to topographic underlying substrata. Contact guidance has also been reported to stimulate growth [18], protein synthesis [19], proteinase secretion [20], and phagocytosis activities [21] in different cell types. Contact guidance of different cell types on a variety of topographic textures was later observed but few quantitative examinations of cell reactions to these topographies were made [9]. When flow was introduced in conjunction with topographic surfaces, there was no noticeable change of cell shape, adhesion, and migration under moderate shear stress [12]. However, when the shear stress was high enough, cell shape, adhesion, and migration was affected by the direction of the flow, and cell attachment would be affected by these shear stresses [7,12,22].

A variety of techniques have been used to create surface topographies for cell scaffolding. These fall into two main categories: techniques that create either ordered or non-ordered topographies [23]. Unordered topographies are generally spontaneously formed during other manufacturing processes in which the organization and orientation of patterns obtained are randomly distributed, the geometry of which cannot be controlled with any precision [23]. Ordered topographies can be fabricated in several ways: photolithography, micro-contact printing, cast moulding, and electron beam lithography are commonly used techniques. All these techniques are able to fabricate ordered topographies but are either time consuming, costly, or both. With the prospect that surface topography influences cell behaviour and increased surface roughness may enhance the adhesion, growth [24] and migration of endothelial cells, we patterned photopolymer films with different groove sizes by a technology called photoembossing [25,26,27], aiming to observe how human umbilical vein endothelial cells (HUVECs) will react on them. 

**Figure 1:**
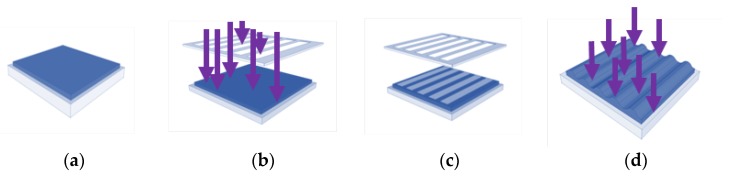
Photoembossing procedure (**a**–**d**); (**a**) film preparation by wire-bar coating; (**b**) UV exposure through a patterned photo-mask; (**c**) removal of photo-mask followed by thermal treatment; (**d**) flood exposure to UV light to cure residual monomer.

Photoembossing is a relatively new technology to create ordered topographies in soft materials, which is both time saving and cost effective. Photoembossing uses a mixture of a multifunctional monomer, a polymeric binder, and a photo-initiator. The photoembossing procedure involves creating a thin film from the mixture followed by ultra violet (UV) light irradiation through a patterned contact photo-mask. The photo-initiator generates free radicals in the areas exposed to UV-light and the monomers in the exposed areas are polymerized upon heating. Depletion of monomer in the illuminated areas creates a monomer concentration and chemical potential difference between the exposed regions and the non-exposed regions which results in the diffusion of monomer from non-exposed to exposed regions. The mass transport that follows from the dark to the illuminated areas will then create a surface relief structure (Figure 1). In photoembossing, there is no mould or etching step required as in other processes.

In a previous paper [27], we mainly evaluated the biocompatibility of photopolymer blends like PMMA-TPETA. Here, we will concentrate on the effect of surface texturing by photoembossing to cell reactions and interactions of these blends.

## 2. Materials and Methods

A polymer binder poly(methyl methacrylate) (PMMA) with a molecular weight of 120,000 g/mol, multifunctional monomer trimethylolpropane ethoxylate (TPETA), photo initiator Irgacure 369 and solvent propylene glycol monomethyl ether acetate (PGMEA) (all obtained from Sigma Aldrich, Gillingham, UK) were used in this study. The CellTiter 96^®^ Aqueous One Solution used in MTS (3-(4,5-dimethylthiazol-2-yl)-5-(3-carboxymethoxyphenyl)-2-(4-sulfophenyl)-2H-tetrazolium) cell proliferation assay was purchased from Promega Corp. (Southampton, UK). Invitrogen CellTracker™ Red CMPTX Probes for Long-Term Tracing of Living Cells were obtained from ThermoFisher Scientific (Paisley, UK).

### 2.1. Grooved PMMA Films by Photoembossing

**Figure 2:**
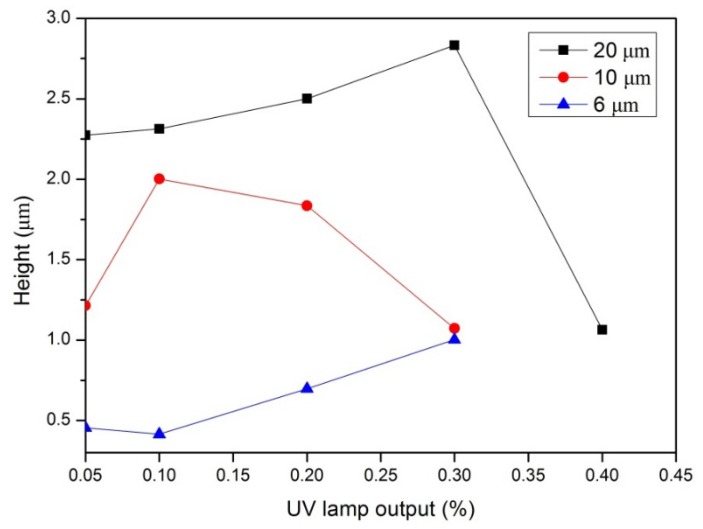
AFM height measurements of photoembossed PMMA-TPETA films as a function of UV dosage for pitches of 6, 10, and 20 µm.

The polymer binder PMMA, TPETA monomer, and biocompatible photo-initiator Irgacure 369 were dissolved in the organic solvent PGMEA. PMMA and TPETA were in the proportion of 50:50 w/w and Irgacure 369 at 5 wt % of TPETA. All components were dissolved in 60 wt % PGMEA. All films were prepared on 13 mm glass cover slips using a wire bar coater which resulted in a thickness of 60 ± 5 µm from which the solvent was evaporated in an oven at 80 °C. To produce smooth films as control substrates, after drying, the films were exposed to direct UV light for 1000 s causing all the monomers to polymerize. To produce surface textured samples, films were covered with a photomask and exposed to UV light for 30 s and then heated for 20 min at 120 °C to accelerate the diffusion of unreacted monomer, and which previous studies indicated as the optimum developing temperature [25]. Moreover, it was shown that there is an optimum UV dosage required to create any particular relief structure [25,26]. Hence, it was necessary to adjust the UV output to create grooves of different heights. To create height structures of 1 µm, the percentage of UV lamp output was set at 30% (522 mJ/cm^2^) for the 6 µm and 10 µm pitch samples, and 20% (344 mJ/cm^2^) and 40% (704 mJ/cm^2^) for 20 µm pitch samples and heights of 2.5 µm and 1 µm, respectively [27]. Finally, the textured films were again exposed to UV light for 1000 s causing the remaining monomers to polymerize. All the samples were dried under vacuum at room temperature for 24 h to evaporate any possible remaining solvent. Different specifications of photomasks and UV dosages were used to produce grooved films with pitches of 6, 10, and 20 µm. Atomic force microscopy (AFM) (NT-MDT^®^, Apeldoorn, The Netherlands) was used to measure the heights at different UV dosages (see Figure 2), with a height of 1 µm being chosen to investigate the influence of pitch size on cell adhesion.

Films with a groove pitch of 20 µm and a height of 2.5 µm were also fabricated to study the effect of groove height at a pitch 20 µm as this showed good biocompatibility in previous studies [27].

### 2.2. Cell Culture

HUVECs (Lonza Group Ltd., Slough, UK) from passages three to seven were grown in M199 medium at 37 °C and 5% CO_2_. The culture medium was supplemented with 10% FBS, 1 ng/mL EC growth factor-β, 3 μg/mL EC growth supplement from bovine neural extract, 1.25 μg/mL thymidine, 10 μg/mL heparin, 100 U/mL penicillin, and 100 mg/mL streptomycin. The medium was changed every two days as previously described [27].

Before cell seeding, the substrates were washed three times with 75% ethanol and rinsed three times with Dulbecco’s phosphate buffered saline (DPBS). When the cells reach around 80% confluency in a T25 (25 cm^2^) cell culture flask, cells were seeded onto the substrate at a density of 1.5 × 10^4^ cells/cm^2^ in 24-well plates and cultured up to seven days for a proliferation assay and 14 days for a wound-healing assay. Untreated glass cover slips were seeded with cells and used as controls.

### 2.3. Cell Proliferation Assay

Cell proliferation on the surfaces of both smooth and grooved PMMA-TPETA films was determined at time intervals of 1, 2, 3, 5, and 7 days. After seven days, some of the samples were confluent—i.e., cells fully covered the substrate. PMMA-TPETA non-embossed films and PMMA-TPETA embossed films with a 20 µm pitch and 2.5 µm height were used for the MTS assay. Untreated 13 mm glass cover slips and cover slips coated with pure PMMA films were used as control. Cell proliferation was measured using an MTS reagent. At each time point, samples were washed two times with serum free culture medium to remove non-adherent cells and then transferred to a new 24-well plate. These samples were then incubated with 50 µL MTS reagent and 250 µL of serum free culture medium for 3 h. Aliquots were put into a 96-well plate and their absorbance was read on a FLUOstar Galaxy Multi-Functional Microplate Reader at 450 nm. Each experiment was repeated at least five times in triplicate.

### 2.4. Analysis of Films and Cells Morphology

**Figure 3:**
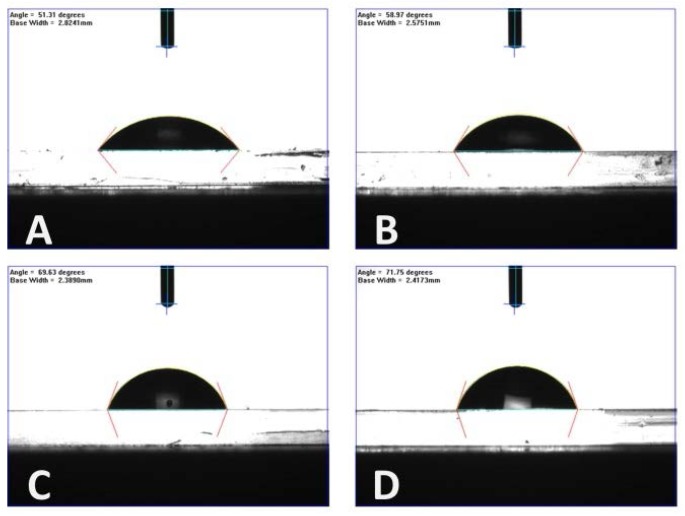
Typical contact angle measurements for each type of PMMA-TPETA film. (**A**) Smooth surface; (**B**) 6 µm pitch; (**C**) 10 µm pitch; and (**D**) 20 µm pitch.

Groove sizes were measured by AFM (NT-MDT^®^) in semi-contact mode at randomly selected locations on a number of samples (*n* ≥ 5). The surface wettability of each film was also measured by a dynamic contact angle system. At least 10 samples were measured for each pitch size, and five measurements were taken for each sample. Figure 3 shows typical measurements for each type of PMMA-TPETA film.

After the cell culture stage, the surface morphology of both smooth and grooved substrates was examined using an ESEM (FEI Inspect-F^®^, Eindhoven, The Netherlands). Following three washes with DPBS, cells were fixed in 4% paraformaldehyde (PFA) for 8 min. After rinsing three times each with DPBS and DI-water, samples were dehydrated by immersing them for 15 min each in a series of increasingly concentrated ethanol solutions (10, 30, 50, 70, 90, 95, and 100%) and then air dried overnight. Dried samples were sputter coated with gold before being examined in an ESEM at a voltage of 10 kV.

### 2.5. Wound-Healing Assay

A wound-healing assay was used to investigate cell migration both on smooth and photoembossed films with different pitches and depths. Grooved films with a groove height of 1 µm and all three pitches (6, 10, and 20 µm) were fabricated for the wound-healing assay. In addition, 20 µm pitch samples but with a groove height of 2.5 µm were also prepared for comparison purposes. After 14 days of culture, cells were confluent on every sample, a scratch was made across each sample surface using a 200 µm wide metal blade after which the medium was replaced with 1 mL fresh complete medium. Since the pressure could not be controlled very accurately, samples with wounds of 200 ± 10 µm in width were selected for the migration experiments with the exception of the 6 µm pitch samples on which the wounds were 150 ± 10 µm. At the start of the experiment (t = 0) and at time intervals 2, 3, and 4 h after the beginning of incubation, samples were stained with CellTracker and analysed under an epifluorescence microscope. At time interval 1 h, there were no significant changes observed, so results are not shown here. Each experiment was repeated at least five times in triplicate.

### 2.6. Immunostaining

HUVECs seeded on 20 µm pitch photoembossed films always seem to fade into the substrates when observed under an optical microscope. To avoid this problem, the cells were stained with the CellTracker™ Red CMPTX and examined under an epifluorescence microscope. The HUVECs were washed three times with culture medium and then stained with the CellTracker for 15 min at room temperature, again washed three times with culture medium and then kept in culture medium with 10% FBS. At the beginning of the experiment (t = 0), cells were stained before being scratched. At the other time intervals, cells were stained before analysis in order to prevent the fluorescence intensity of the samples from being quenched. Due to the bleaching of the samples during examination, different samples were used for each group at different time intervals. Experiments were repeated five times and each time samples were prepared in triplicate.

### 2.7. Data Analysis and Statistical Methods

All quantitative measurements are reported as average values ± standard deviation. To determine the statistical difference between two mean values, a student’s *t*-test was used to analyze the two different materials at similar time intervals.

## 3. Results

### 3.1. Synthesis and Characterization of Photoembossed PMMA-TPETA Films

Photoembossed PMMA-TPETA films with pitches of 6, 10, and 20 µm were successfully fabricated similar to [27]. Moreover, we can see from the contact angle results (Table 1) that all PMMA-TPETA films are hydrophilic. However, as the pitch size of the relief structure increases, the contact angle also gets slightly larger. This means that the surfaces with smaller texture sizes are more hydrophilic. Nevertheless, we do not consider the differences between textures of 10 µm and 20 µm to be significant.

### 3.2. HUVEC Proliferation and Morphology

Previous studies have indicated that a contact angle of around 70° is most suitable for cell adhesion [28], suggesting optimal cell adhesion for photoembossed films with a pitch of 10 µm or 20 µm. This was confirmed by the following MTS assay. It should be noted that contact angle measurements did not show any significant asymmetric wetting for samples of the same material and surface texture.

**Figure 4:**
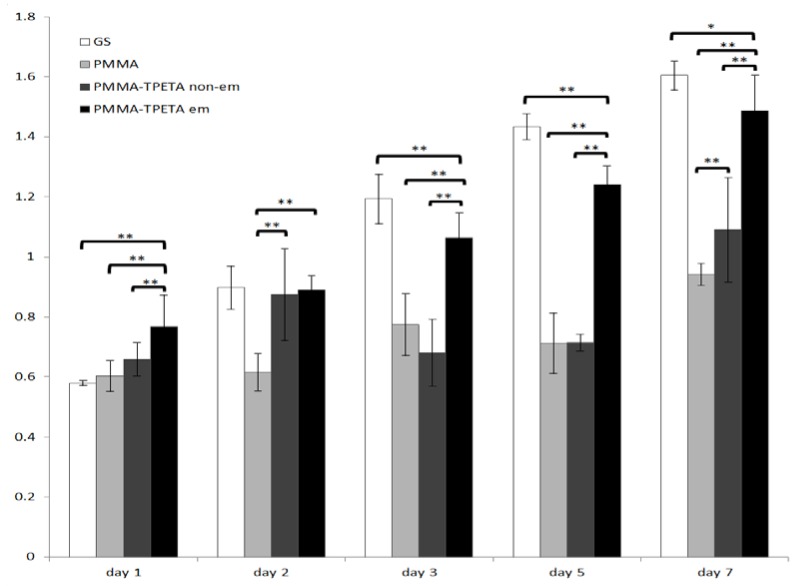
Proliferation of HUVECs on glass cover slips, pure PMMA films, non-embossed PMMA-TPETA films, and photoembossed PMMA-TPETA films at Day 1, 2, 3, 5, and 7. * indicates statistical significance with *p* < 0.05 and ** indicates significance with *p* < 0.01.

The MTS results (Figure 4) showed that cell metabolic activity for all samples had increased by the end of the time period studied. To be specific, cell metabolic activity on pure PMMA films and PMMA-TPETA non-embossed films dropped on the fifth and third day, respectively, but increased later. However, cells seeded on the embossed films grew steadily throughout the experimental period and showed significantly higher proliferation compared with the other two sample categories at the end of the experiment. Cells on embossed PMMA-TPETA films showed significantly higher proliferation compared with those on pure PMMA films and significantly higher proliferation than on non-embossed surface for the majority of the experimental period, but especially from Day 3 onwards. Cells on non-embossed PMMA-TPETA films also showed significantly higher proliferation than those on pure PMMA films by the end of the experiment. These results suggest that the photopolymer improved the biocompatibility of PMMA, while the grooved texture greatly enhanced cell proliferation. Additionally, confluence cells are still in good condition when they are used for the cell migration assay after up to 14 days’ culture.

**Figure 5:**
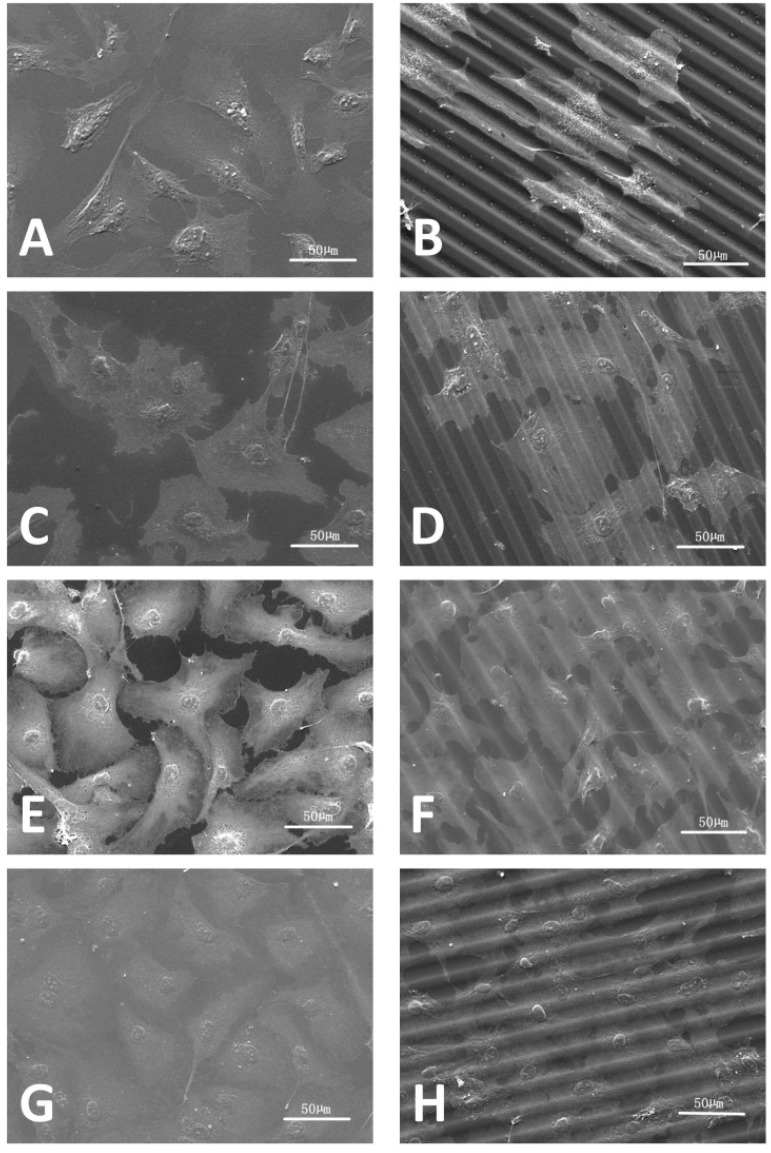
HUVECs seeded on non-embossed (**left**) and embossed PMMA-TPETA films (**right**), respectively, at Day 1 (**A**,**B**), Day 3 (**C**,**D**), Day 5 (**E**,**F**), and Day 7 (**G**,**H**).

On embossed PMMA-TPETA films, clear cell alignment along the surface relief texture is observed at Day 1 (Figure 5B), while cells organised themselves randomly on smooth surfaces (Figure 5A). As the culture time increased, cells became more flattened and dispersed on all substrates. The ESEM pictures also show a reduced cell density on non-embossed PMMA-TPETA films at Day 3 (Figure 5C), which is in accordance with the MTS results. Also by Day 3, cells begin to show a tendency to bridge the relief structures and less alignment can be seen (Figure 5D). By Day 7, cells almost fully cover the surface of the substrates on both non-embossed (Figure 5G) and embossed (Figure 5H) film samples. However, it seems that by providing a larger surface area, the grooved films allowed more cells to attach. Next to patterning, this enlarged surface area could be another reason why, by the end of the assay, cell proliferation on embossed films is significantly higher than on non-embossed surfaces. However, as surface texturing and enlarged surface area are happening at the same time, it is difficult to separate these effects.

### 3.3. Cell Migration

**Figure 6:**
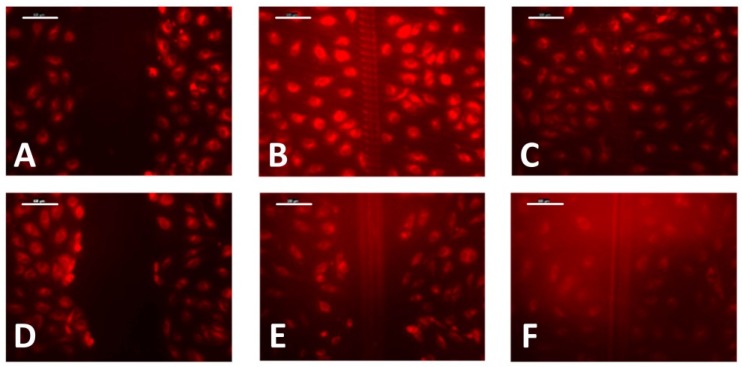
Wound-healing assay on photoembossed (**top**) and smooth (**bottom**) films at time intervals of 0 h (**A**,**D**), 2 h (**B**,**E**), and 3 h (**C**,**F**). Scale bar = 100 µm.

The non-embossed PMMA-TPETA films were first compared with embossed PMMA-TPETA films with 20 µm pitches and 2.5 µm heights (Figure 6) in a wound-healing assay. The results show that after 2 h, there is a noticeable difference between these two samples and after 3 h, cells on the textured film have almost filled the wound, whereas cells on the smooth film still show a significant gap at the wound site. 

**Figure 7:**
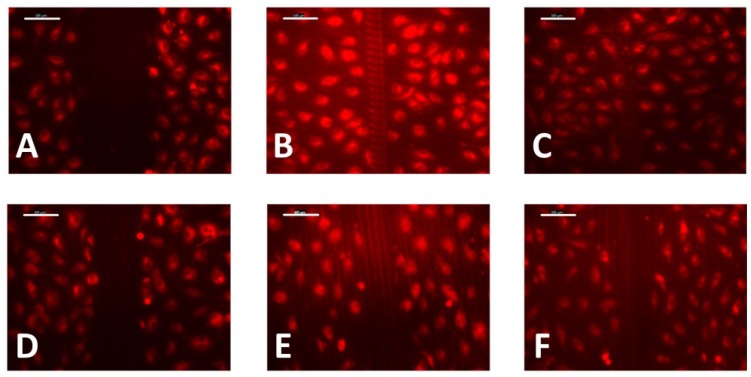
Wound-healing assay on photoembossed films with perpendicular (**top**) and parallel (**bottom**) wounds to the direction of the grooves at time intervals of 0 h (**A**,**D**), 2 h (**B**,**E**), and 3 h (**C**,**F**). Scale bar = 100 µm.

On films embossed with grooves with a 20 µm pitch and 2.5 µm height, a comparison was made between wounds made perpendicular to and parallel with the grooves (Figure 7). Using approximately the same pressure to scratch the wound, the wounds made in a parallel direction were initially significantly narrower and differences between the perpendicular and parallel samples were still apparent after 3 h. These results indicate that grooves seem to inhibit cell migration when wounds are made parallel to the grooves. On the other hand, comparatively smaller wounds made parallel to the grooves indicate that, in this case, cells are less likely to detach from the substrate.

**Figure 8:**
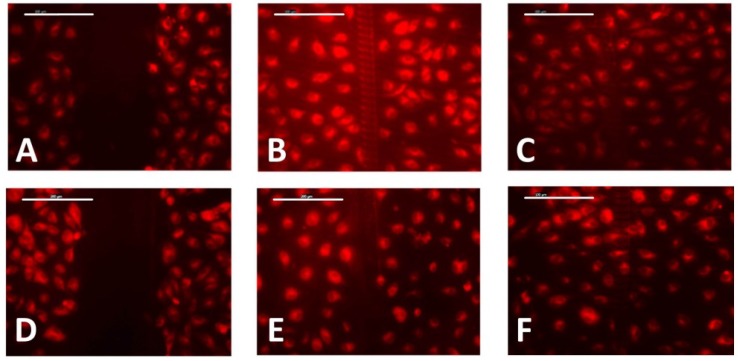
Wound-healing assay on photoembossed films with 2.5 µm pitch (**top**) and 1 µm pitch (**bottom**) at time intervals of 0 h (**A**,**D**), 2 h (**B**,**E**), and 3 h (**C**,**F**). Scale bar = 200 µm.

Photoembossed PMMA-TPETA films with a 20 µm pitch and heights of 2.5 µm and 1 µm are compared in Figure 8. The results indicate no significant differences in initial wound sizes, cell migration speed, and wound recovery time.

**Figure 9:**
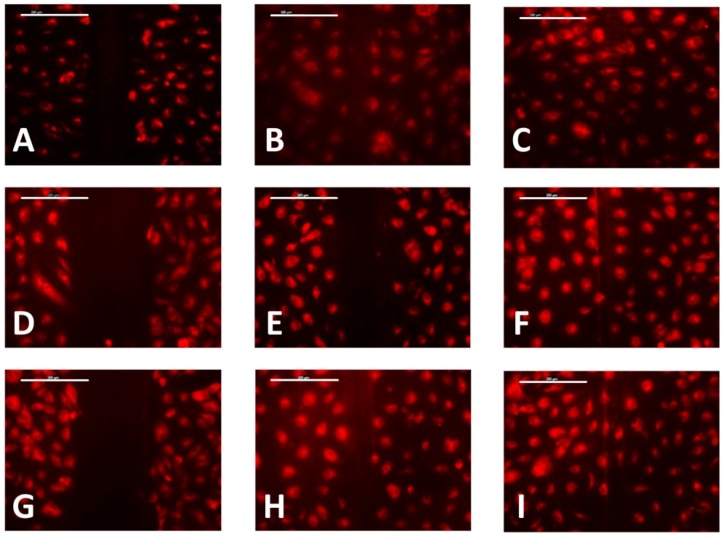
Films with 6 µm (**top**), 10 µm (**middle**) and 20 µm (**bottom**) pitches and 1 µm height at time intervals of 0 h (**A**,**D**,**G**), 2 h (**B**,**E**,**H**), and 4 h (**C**,**F**,**I**). Scale bar = 200 µm.

A wound-healing assay on films with 6, 10, and 20 µm pitches, all with a ridge height of 1 µm were also compared (Figure 9). The results indicate that for scratches made on embossed films at a similar pressure, wounds appeared significantly narrower for samples with a 6 µm pitch compared with 10 µm and 20 µm pitch samples. After 2 h, the wounds on the 20 µm pitch samples were clearly narrower than on the 10 µm pitch samples and were similar in size to those on 6 µm samples, which as mentioned earlier, were initially narrower. However, all the wounds had recovered after 4 h.

**Figure 10:**
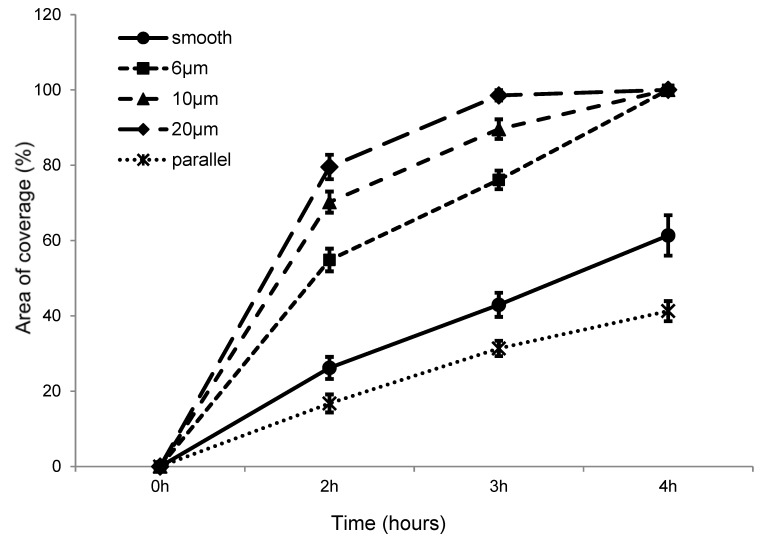
The percentage of the recovered area on smooth PMMA-TPETA films and embossed films with 6, 10, and 20 µm pitches with the direction of grooves perpendicular to the wound. A comparison is also made with a photoembossed film with 20 µm pitches with the direction of grooves parallel to the wound.

Figure 10 summarizes the percentage of the recovered area for samples with different surface topology at every time interval. As there were only minor changes in the first hour, observations at this time interval are not shown. However, from the time intervals 2–4 h, we can still see that the cells migrate in nearly one direction.

## 4. Discussion

PMMA has proven to exhibit good biocompatibility and is in wide clinic use, such as in rigid intraocular lenses [29], bone cement [30], dental filling, dentures [31] and cosmetic surgery [32]. Here, we demonstrated that by adding a biocompatible monomer TPETA, the resultant photopolymer has increased biocompatibility compared to pure PMMA.

Photoembossing is a relatively new technology for the fabrication of surface textures. Compared to photolithography, which is the most commonly used method to pattern thin films or substrates by microfabrication, photoembossing does not require a wet-etching step which makes it more cost-effective and environmental friendly. In this method, the shape of the relief structures is determined by the pattern of the photomask while our previous work has confirmed that the depth of the relief structures can be adjusted by controlling the UV dosage and temperature during the heating stage [25,27]. By this process of photoembossing and controlled UV dosages, we successfully fabricated photopolymer films with 1 µm high relief structures and 6, 10, and 20 µm pitches, as well as films with 2.5 µm high microstructures with a 20 µm pitch.

The changes in surface morphology of these embossed films leads to changes in wettability. Embossed films are less hydrophilic than smooth films. However, 6 µm pitch films are more hydrophilic than 10 µm or 20 µm pitch films, while the latter two are comparable in wettability. According to Van Wachem et al. [33], a substrate which is too hydrophilic or too hydrophobic is less desirable for attachment and proliferation of HUVECs. Similar results have been found for other cell types and substrate materials [34,35,36,37,38,39]. A comparison of the MTS assay results for non-embossed and embossed PMMA-TPETA films indicate that the initial attachment of HUVECs on embossed films is significantly greater than on non-embossed films. Additionally, the proliferation of the cells on embossed films is significantly greater than on smooth films from Day 3 onwards. This suggests that decreased wettability due to surface texturing by photoembossing promotes adhesion and proliferation of HUVECs in these experiments.

PMMA-TPETA photopolymer shows improved biocompatibility compared to pure PMMA which has already been recognized as a polymer with a good level of biocompatibility and which is already widely used in the medical sector. However, grooved surfaces significantly promoted cell proliferation, and due to their enlarged surface area, enabled the accommodation of more cells than smooth surfaces.

**Figure 11:**
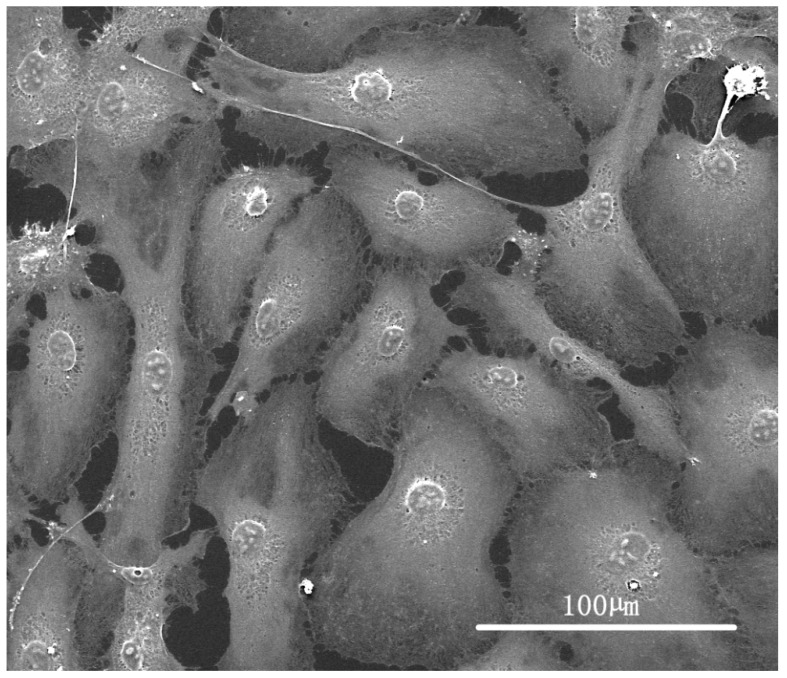
ESEM picture of HUVECs in vitro.

Cells showed the most alignment on Day 1 and flatten and disperse as time passes. There could be several reasons for this. First, cell orientation depends on cell type. HUVECs cultured in vitro are polygonal cells with a width between 30–50 µm and tend to return to this shape [40] (Figure 11). Secondly, as the culture proceeds, contact inhibition between cells becomes stronger and might even become the dominant factor in affecting the cell shape rather than the effect of contact guidance from the surface topography. Additionally, fibronectin (Fn), a protein considered to promote cell adhesion, contained in FBS deposits on the surface of the substrate may also weaken the influence of contact guidance on the cells.

In a conventional wound-healing assay on well plates, the average migration rate is 25 µm/h [41]. The results of our wound-healing assay presents significantly accelerated recovery speeds with uniform motion, HUVECs with 200 µm wounds are fully recovered after 4 h on all embossed films with 6, 10, and 20 µm pitches, while wounds on the smooth films are still clearly visible. Additionally, 20 µm appears to be the most efficient among the three examined pitches while there was no noticeable difference between samples with relief heights of 1 µm and 2.5 µm. On 20 µm embossed films, the wound-healing assay was also conducted for wounds perpendicular to and parallel with the relief structures. The results show that, after 3 h, the wound perpendicular to the surface texturing has fully recovered, while the wound parallel to the texturing remains largely unaffected. These results suggest that, with the exception of this last case of a ‘parallel wound’, all the grooved surface textures and variations of different pitch sizes that were tested accelerate cell migration, with 20 µm pitch grooves performing the most effective. Moreover, films with 10 µm and 20 µm pitches also have water contact angles of around 70°, which according to Tamada and Ikada [28] are not only optimal for cell adhesion but in our case may also accelerate cell migration. However, a grooved surface texture can also inhibit cell migration when the wound is parallel to the groove direction, although at the same time it can prevent the cells from detaching from the substrates. Additionally, when the pitch of the surface texture is as low as 6 µm, cells are again protected from detaching.

## 5. Conclusions

Photoembossing is a simple, environmentally friendly, and cost-effective way to fabricate surface topographies in polymer films. Here for the first time we reported on the responses of cells to patterned substrates produced by photoembossing. Interestingly, the photopolymer PMMA-TPETA showed improved biocompatibility compared to neat PMMA, offering great potential for applications in cell and tissue engineering. Surface texturing by photoembossing decreased the surface hydrophilicity of polymer films and promoted cell attachment, proliferation, and migration. Cell alignment was affected by the surface relief structure most distinctly at the beginning of culture. In a wound-healing assay, photoembossed surfaces accelerated the migration of HUVECs along the polymer film structure, with the cells migrating relatively more quickly on embossed films with larger pitches, having a contact angle of around 70°. Grooved surface textures with ‘parallel wounds’ and a pitch of 6 µm also inhibited cell detachment. All these results suggest that photoembossed PMMA-TPETA films can be used to develop a monolayer of HUVECs on the surface of such films for applications in artificial vascular implants, preventing the formation of thrombi and neointima.

## Figures and Tables

**Table 1:** Contact angles of PMMA-TPETA films with different surface topographies.

PMMA-TPETA Films	Contact Angle (Degrees)
Smooth	50 ± 1.6
6 µm pitch	59 ± 1.4
10 µm pitch	70 ± 0.3
20 µm pitch	71 ± 0.9
